# Newly detected rapid eye movement associated sleep apnea after coronavirus disease 2019 as a possible cause for chronic fatigue: two case reports

**DOI:** 10.1186/s13256-021-02819-0

**Published:** 2021-04-22

**Authors:** Andreas Rembert Koczulla, Antje Stegemann, Rainer Gloeckl, Sandra Winterkamp, Bernd Sczepanski, Tobias Boeselt, Jan Storre, Michael Dreher

**Affiliations:** 1 Department of Pulmonary Rehabilitation, University Medical Center Giessen and Marburg, Philipps-University Marburg, Member of the German Center for Lung Research (DZL), Marburg, Germany; 2 Institute for Pulmonary Rehabilitation Research, Schoen Klinik Berchtesgadener Land, Malterhoeh 1, 83471 Schoenau am Koenigssee, Germany; 3 Teaching Hospital, Paracelsus Medical University, Salzburg, Austria; 4 Department of Pulmonology, Philipps-Universität Marburg, Marburg, Germany; 5 Pneumologie Solln, Munich, Germany; 6 Department of Pneumology, University Medical Hospital, Freiburg, Germany; 7 Department of Pneumology and Intensive Care Medicine, University Hospital Aachen, Aachen, Germany

**Keywords:** COVID-19, Sleep apnea, REM phase, Fatigue syndrome, APAP, CPAP, Case report

## Abstract

**Background:**

Coronavirus disease 2019 has become a health problem spreading worldwide with pandemic characteristics since March 2020. Post coronavirus disease 2019 symptoms are more frequent than initially expected, with fatigue as an often-mentioned issue.

**Case presentations:**

We describe a 32-year-old white male and a 55-year-old white female who suffered from post coronavirus disease 2019 fatigue syndrome. On polysomnography, rapid eye movement associated sleep apnea with an increased hypopnea index during rapid eye movement phases of 36.8 and 19.5 events per hour was found. Based on the patients’ burdensome fatigue symptoms, we initiated automatic positive airway pressure therapy, which diminished sleep apnea (rapid eye movement index: 0.0 in both patients) and, consequently, also the fatigue symptoms.

**Conclusions:**

Since sleep apnea and coronavirus disease 2019 are both associated with fatigue, a screening for sleep apnea might be considered in coronavirus disease 2019 patients with fatigue syndrome.

## Background

The coronavirus disease 2019 (COVID-19) pandemic spread rapidly worldwide in early 2020. According to the German Robert Koch Institute, a person is considered to be recovered from COVID-19 if he or she does not show any symptoms (such as cough or fever) for at least 48 hours in combination with a negative nasopharyngeal swab test. However, it is not uncommon that secondary COVID-19 symptoms remain after the acute infection phase [1]. These include but are not limited to fatigue syndrome, cognitive impairments, or reduced stress management. Fatigue syndrome is particularly burdensome for many patients after surviving a COVID-19 infection. This applies to patients that received outpatient care as well as to patients that were hospitalized [2].

Case presentation #1

A 32-year-old white physician without any previous disease got infected with SARS-CoV-2 in April 2020. The patient is of athletic condition with a normal to slightly reduced body mass index (20.1 kg/m^2^) and had never reported fatigue symptoms, concentration problems, or cognitive impairment before his SARS-CoV-2 infection. Although the acute symptoms of the COVID-19 phase had eased during a recovery phase at home, the patient still perceived limitations of physical performance and reported difficulties in concentration. In addition, the patient described severe general fatigue symptoms following COVID-19 as the predominant burden. Since these symptoms persisted, the patient was referred to a pulmonary rehabilitation program at the Schön Klinik Berchtesgadener Land (Germany) in August 2020.

**Fig. 1:**
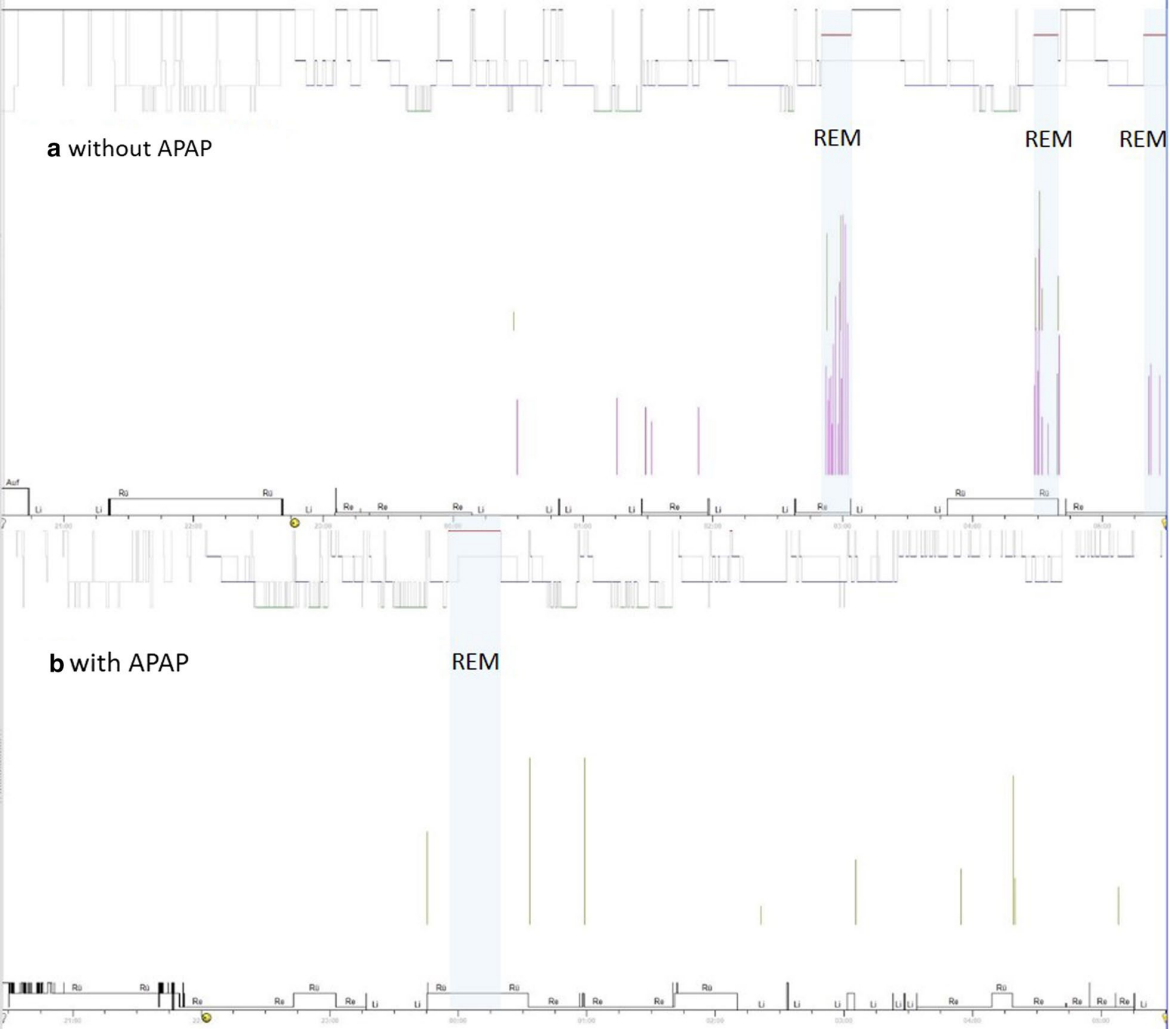
Polysomnography of case 1 without automatic positive airway pressure (**a**) and with automatic positive airway pressure (**b**). Pink peaks indicate events of REM-associated sleep apnea

At baseline assessment, the patient had normal lung function [FEV_1_: 118% predicted, total lung capacity (TLC): 99% predicted, VC: 99% predicted] and diffusion capacity (DLCO: 101% predicted). Computed tomography of the thorax was without pathological findings. Regarding the fatigue syndrome, fasting cortisol as well as thyroid parameters were analyzed but found to be within normal ranges. Therefore, we decided to perform a polysomnography for further clarification. An increased apnea–hypopnea index (AHI) of 5.2 was observed only during the rapid eye movement (REM) phases including 36.8 hypopnea events per hour (Fig. 1). This sleep apnea led to nocturnal awakening of the patient with a concomitant sensation of choking. We associated this sleep apnea with the fatigue syndrome of the patient. As a therapeutic consequence, automatic positive airway pressure (APAP) therapy was initiated. The patient showed good adherence to the APAP therapy using a nasal pillow mask with a pressure support between 5 and 7 mbar.

After 11 days of APAP therapy, the polysomnography was repeated and showed that sleep apnea has disappeared completely (AHI 0.0, and 0 hypopnea events; Table 1 and Fig. 1). Besides, the patient reported improvements in his fatigue and the ability to concentrate.

**Table 1:** Parameters during polysomnography without and with the use of automatic positive airway pressure therapy in two cases after post COVID-19

	Case 1		Case 2	
Parameter	Without APAP	With APAP	Without APAP	With APAP
Apnea–hypopnea index	5.2	0.0	6.2	0.0
Number of hypopnea events during REM phase	22	0	13	0
Number of hypopnea events during non-REM phase	6	0	8	1
Hypopnea index during REM phase (events/hour)	36.8	0.0	19.5	0.0
Hypopnea index during non-REM phase (events/hour)	1.2	0.0	1.4	0.2
Maximum duration of hypopnea during REM phase, seconds	54.0	0	26.5	0
Maximum duration of hypopnea during non-REM phase, seconds	29.0	0	29.5	17.0
Mean duration of hypopnea during REM phase, seconds	25.7	0	15.2	0
Mean duration of hypopnea during non-REM phase, seconds	16.6	0	13.0	17.0
Arousals, *n*	137	48	46	43
SpO_2_ nadir during REM phase, %	90	96	85	88
Maximum heart rate during REM phase, beats per minute	68	60	77	68

*APAP* automatic positive airway pressure, *REM* rapid eye movement, *SpO*_*2*_ oxygen saturation

Case presentation 2

A 55-year-old healthy white nurse got infected with SARS-CoV-2 in April 2020. The patient is of slightly obese condition with an increased body mass index (31.0 kg/m^2^) but had never reported fatigue symptoms, cognitive impairments, or sleep disorders before her SARS-CoV-2 infection. Although the acute symptoms of the COVID-19 phase had eased during a recovery phase at home, the patient still perceived ongoing severe general fatigue symptoms as the predominant burden. In addition, the patient reported difficulties in memory performance and amnestic dysphasia.

On admission to pulmonary rehabilitation in September 2020, the patient had almost normal lung function (FEV_1_: 91% predicted, TLC: 90% predicted, VC: 86% predicted) and diffusion capacity (DLCO: 90% predicted). Spiroergometry revealed slightly reduced exercise performance (87% predicted) but no evidence for cardioventilatory limitations or impairment in lung diffusion capacity during exercise. Further, a polysomnography was performed for further clarification. An apnea–hypopnea index (AHI) of 6.2 was observed only during the REM phases including 19.5 hypopnea events per hour. This sleep apnea led to nocturnal awakening of the patient associated with a burning sensation in the lungs. As a therapeutic consequence, automatic positive airway pressure (APAP) therapy was again initiated. The patient showed very good adherence to the APAP therapy using a full face mask with pressure support between 7 and 10 mbar.

After 7 days of APAP therapy, the polysomnography was repeated and showed that sleep apnea had disappeared almost completely (AHI 1.7, and 0 hypopnea events; Table 1). Furthermore, the patient reported improvements of her fatigue symptoms as well as her concentration and memory performance.

Discussion and conclusions

To the best of our knowledge, these are the first two reported cases of fatigue following COVID-19 that might be induced by sleep apnea during the REM phase. The patients suffered from self-reported severe general fatigue and concentration impairment. These clinical symptoms as well as the sleep apnea were diminished after initiating APAP therapy.

Both patients had no history of sleep apnea or any other risk factors (except for slight obesity in patient 2). Therefore, the sleep apnea events that occurred during the non-REM phases could have been associated with mild obesity hypoventilation syndrome without clinical relevance since the clinical symptoms of fatigue and cognitive impairment appeared only after SARS-CoV-2 infection. Interestingly, in both cases, sleep apnea occurred only during the REM phases. This is a phenomenon that is not common in other sleep-related disorders. It was shown in former studies that a SARS-CoV-2 infection leads to endotheliitis with immunogen-triggered embolisms [3]. Abnormalities were found also in neuroimaging, which revealed diffuse cerebral hyperintensities that are indicative of leukencephalopathy [4] and perfusion abnormalities [5]. Up to now, ischemic stroke is the only disease that is known to depress REM sleep [6]. Therefore, endotheliitis-induced perfusion defect might be an individual explanation for the disturbed sleeping profile in the current COVID-19 cases. However, this hypothesis needs to be investigated in further research.

In conclusion, in the current two cases, newly diagnosed sleep apnea during the REM phases with subsequent severe fatigue syndrome was observed following COVID-19. APAP therapy diminished sleep apnea completely. Furthermore, patients reported that the post-COVID-19 fatigue symptoms and their ability to concentrate have improved.

Though it is possible that COVID-19 infection and subsequent diagnosis of sleep apnea are unrelated and do not suggest causality, sleep apnea must be considered in the differential diagnosis of patients with post-COVID-19 fatigue syndrome.

## Data Availability

The datasets used during the current study are available from the corresponding author on reasonable request.

## Abbreviations

AHI: Apnea–hypopnea index
APAP: Automatic positive airway pressure
DLCO: Diffusion lung capacity for carbon monoxide
FEV_1_: Forced expiratory volume in 1 second
REM: Rapid eye movement
SARS-CoV-2: Severe acute respiratory syndrome coronavirus 2
SpO_2_: Oxygen saturation
TLC: Total lung capacity
VC: Vital capacity
